# Frictional characterization of injectable hyaluronic acids is more predictive of clinical outcomes than traditional rheological or viscoelastic characterization

**DOI:** 10.1371/journal.pone.0216702

**Published:** 2019-05-10

**Authors:** Edward D. Bonnevie, Devis Galesso, Cynthia Secchieri, Lawrence J. Bonassar

**Affiliations:** 1 Sibley School of Mechanical and Aerospace Engineering, Cornell University, Ithaca, NY, United States of America; 2 Fidia Farmaceutici, Padua, Italy; 3 Meinig School of Biomedical Engineering, Cornell University, Ithaca, NY, United States of America; University of Rochester, UNITED STATES

## Abstract

Hyaluronic acid injections have been a mainstay of arthritis treatment for decades. However, much controversy remains about their clinical efficacy and their potential mechanism of action. This approach to arthritis therapy is often called viscosupplementation, a term which is rooted in the elevated viscosity of the injected solutions. This terminology also suggests a mechanical pathway of action and further implies that their efficacy is dependent on viscosity. Notably, previous studies of the relationship between viscous properties of hyaluronic acid solutions and their clinical efficacy have not been definitive. Recently we developed an experimental and analytical framework for studying cartilage lubrication that captures the Stribeck-like behavior of cartilage in an elastoviscous transition curve. Here we apply this framework to study the lubricating behavior of six hyaluronan products currently used for injectable arthritis therapy in the US. Despite the fact that the source and chemical modifications endow these products with a range of lubricating properties, we show that the lubricating effect of all of these materials can be described by this Stribeck-like elastoviscous transition. Fitting this data to the elastoviscous transition model enables the calculation of effective lubricating viscosities for each material, which differ substantially from the viscosities measured using standard rheometry. Further we show that while data from standard rheometry are poor predictors of clinical performance of these materials, measurements of friction coefficient and effective lubricating viscosity correlate well (R^2^ = 0.77; p < 0.005) with assessments of improved clinical function reported previously. This approach offers both a novel method that can be used to evaluate potential clinical efficacy of hyaluronic acid formulations and provide new insight on their mode of action.

## Introduction

Intra-articular injections of hyaluronic acid has been a mainstay of arthritis treatment since initial trials of such therapies more than 30 years ago [1,2]. This course of therapy was termed “viscosupplementation” based on the restoration of viscosity observed after delivery to pathologic synovial fluid [3]. Significant controversy persists around the clinical efficacy and proposed underlying mechanisms of these therapies. Recently, conflicting consensus statements were released from multiple clinical societies based on meta-analyses of clinical studies, which show either no statistical effect of hyaluronic acid injections over placebo [4], or beneficial effects at reducing pain and restoring function in cases of mild to moderate osteoarthritis [5,6], particularly in patients with knee OA who have not had an adequate response to non-pharmacologic modalities and full-dose acetaminophen [7–9]. Despite this controversy, use of such products is widespread, and annual sales are expected to surpass $2.6 billion in the near future [10].

This controversy is rooted in the fact that it is difficult to prove clinical efficacy versus placebo and that there is no consensus concerning the mechanism of action. Many studies report that hyaluronic acid has a protective effect on cartilage explants and chondrocytes through multiple biological mechanisms. As a critical component of the cartilage extracellular matrix, hyaluronic acid interacts with chondrocytes through the CD44 receptor [11]. This binding is thought to be both anabolic and anti-catabolic, inhibiting expression and activity of inflammatory cytokines [12] and degradative proteinases in vitro [13], and reducing matrix damage [14], fibrosis [15], and expression of inflammatory markers in vivo [16]. Although such potential biological mechanisms exist, the FDA classifies hyaluronic acid injections as class III medical devices, implying that a primary mode of action is mechanical. Indeed, the lubricating role of hyaluronic acid in synovial fluid has been known for decades [17]. Addition of hyaluronic acid is known to lower friction coefficients of whole joints [18,19] and in ex vivo studies of cartilage-on-cartilage [20] and cartilage-on-glass [21] interfaces. Although injecting hyaluronic acid is commonly referred to as ‘viscosupplementation’, there has been relatively little direct investigation of the extent to which the viscosity of hyaluronic acid governs its lubricating ability and clinical efficacy.

Many studies, focusing on either lubricating properties or in vivo efficacy, have compared hyaluronic acid solutions based on molecular weight, which is typically related to intrinsic viscosity via a power law relationship (i.e., the Mark-Houwink equation). However, inferring the effect of viscosity from such studies can be challenging, as the Mark-Houwink coefficients for hyaluronic acid solutions vary with molecular weight [22]. Further, the relationship between molecular weight and intrinsic viscosity can change when the molecule is modified or partially crosslinked. While molecular weight correlates with improved lubrication in experiments using isolated cartilage tissue [20,23] and whole joints [18], the relationship between molecular weight and clinical outcomes is less clear. Several studies report that high molecular weight formulations improve outcomes, both in preclinical studies [24–26] and in human trials [27]. In contrast, several studies report no clinical benefit of increasing molecular weight [28–34] or crosslinking [35], while others suggest that low molecular weight formulations may be superior [36] due to their ability to more effectively penetrate the cartilage matrix [37]. Additionally, the relationship between rheological and viscoelastic properties and clinical outcomes is complicated due to the complex mechanical properties of HA formulations. Although it is well understood that these solutions are described by non-Newtonian, shear thinning behavior [22], their mechanical properties are not be fully captured by conventional rheology. Recent evidence suggests that standard rheologic analyses are confounded due to the interfacial viscosity [38], and consequently, the in vivo situation is even further confounded as HA is known to interact with the proteins at the articular surface [21,39,40]. Because of these factors, the relationship between HA mechanical properties and clinical efficacy has not been well established.

The lack of clarity from these above studies suggests that a new framework is needed to understand the action of hyaluronic acid and to develop tools to predict both lubricating ability and clinical outcomes. Several studies have attempted to understand cartilage tissue and joint lubrication in the context of classic lubrication framework using a Stribeck curve [19,41,42], where distinct lubrication modes are mapped as a function of sliding speed, normal load, sample geometry, and lubricant viscosity. Classically, for hard permeable materials in specific geometries, the curve maps a frictional transition from boundary mode marked by solid-solid contact, to partial separation of surfaces by pressurized fluid, to full separation by a fluid film, which results in extremely low friction. Recently, it was shown that soft, permeable materials [43], including articular cartilage [21] undergo a similar “elastoviscous transition” in lubrication behavior. Using this framework, the transition of cartilage tissue through different lubrication modes was shown to be predicted by the viscosity of hyaluronic acid solutions.

The goals of the current study were to evaluate the lubricating properties of 6 hyaluronan formulations that are currently used in the US and to determine the extent to which their rheological and viscoelastic properties measured by traditional means, and frictional properties are correlated with their clinical function. Here, we show that commercial formulations of hyaluronic acid produce speed-dependent friction of cartilage, but these friction coefficients are not well described by standard rheological techniques. Further, using a Stribeck-like framework enables the calculation of an effective lubricating viscosity for each formulation that is distinct from the measured dynamic viscosity. Finally, we show that the effective lubricating viscosity and friction coefficient measured in vitro correlate with changes in clinical outcome data aggregated from previous clinical trials.

## Results

### Clinically approved HA formulations exhibit a wide range of rheological behavior

**Fig 1:**
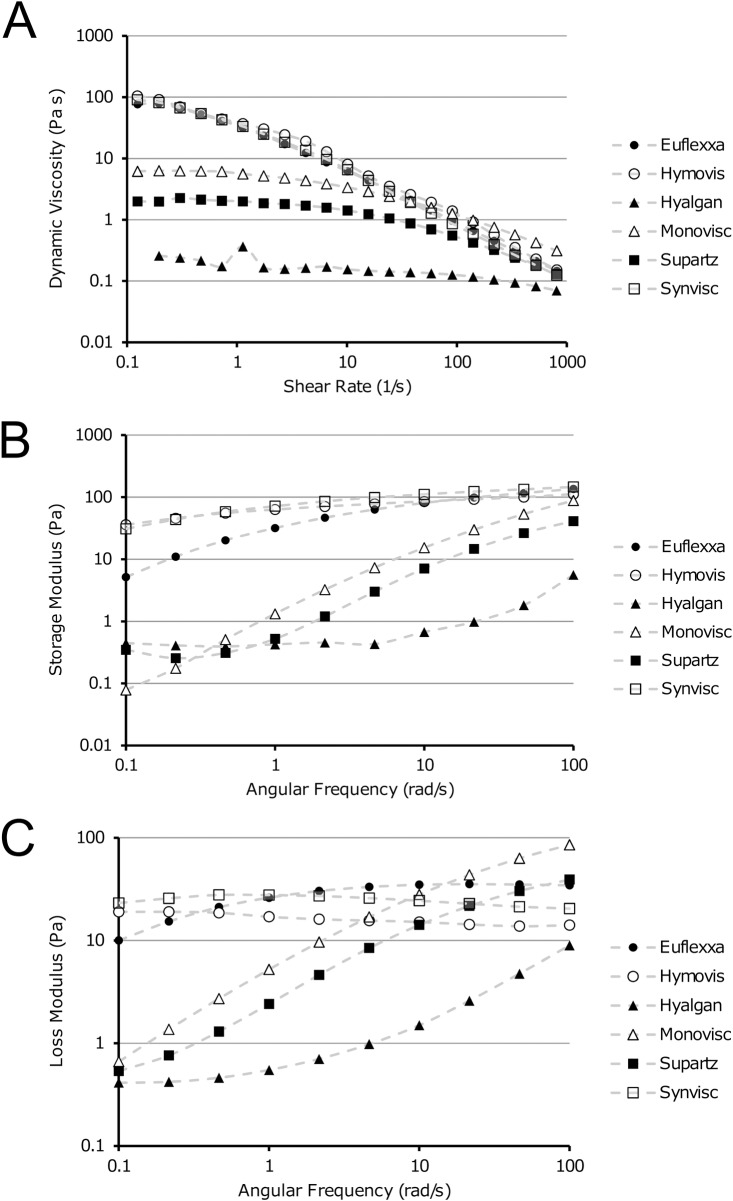
Rheology of clinical HA formulations. (A) Flow sweep experiments revealed shear thinning behavior of all HA formulations, but orders of magnitude variations in dynamic viscosity were evident. (B,C) Storage and loss moduli of the same HA formulations as a function of angular frequency.

To test the hypothesis that the clinical efficacy of HA injections is related to its mechanical properties, we tested six clinically-approved HA formulations (Synvisc, Monovisc, Hyalgan, Euflexxa, Supartz, and Hymovis) using a commercial rheometer. In brief, we conducted experiments in which shear rate was increased from 0.1 to 100 s^-1^ using a cone-plate rheometer configuration. As expected, we found shear thinning behavior in each of the formulations, but viscosities varied by orders of magnitude between the products (**Fig 1A**). To more fully characterize these results, we fit this rheological data to a Carreau-Yasuda model given by the equation:

$$\frac{\eta_{}-\eta_{\infty }}{\eta_{0}-\eta_{\infty }}={\left[1+(\lambda {\dot{\gamma })}^{a}\right]}^{n-1/a}$$ (Eq 1)

Where, *η* is dynamic viscosity, *η*_*∞*_ is the dynamic viscosity at infinite shear rate, *η*_*0*_ is the dynamic viscosity at zero shear rate, $\dot{\gamma }$ is shear rate, and *λ*, *n*, and *a* are fitting parameters (**S1 Fig**). Data from all commercial formulations were described well by the Carreau-Yasuda model, with coefficients of variation <10% for all fits (**Table 1**). Additionally, we characterized the viscoelastic properties of each of these products in oscillatory shear (3% strain from 0.1 to 100 rad/sec). Overall, we found that the rheological properties of all of these HA formulations varied by orders of magnitude. Specifically, the storage and loss moduli ranged from under 0.1 Pa to over 100 Pa and under 1 Pa to 100 Pa, respectively. Additionally, there was a range of phase angles observed at low shear rates (0.1 rad/s) that ranged from 28° to 83° indicating that the HA formulations exhibit a range of elastic versus viscous behavior (**Table 2**).

**Table 1:** Carreau-Yasuda model parameters for 6 clinically approved HA viscosupplements. Interestingly, the zero shear viscosities (i.e., η_0_) of these formulations spanned more than two orders of magnitude.

Lubricant	η_0_ (Pa·s)	η_∞_ (Pa·s)	λ	a	n	CV(RMSD)
Euflexxa	100.09	0.45	1.91	0.94	0.02	0.08
Hymovis	190.37	0.00	13.16	0.40	0.39	0.08
Hyalgan	0.50	0.00	2117.20	0.60	0.88	0.09
Monovisc	6.46	0.00	0.25	0.60	0.48	0.03
Supartz	2.11	0.00	0.09	0.60	0.39	0.05
Synvisc	124.85	0.00	3.86	0.60	0.21	0.03

**Table 2:** Viscoelastic properties of the HA formulations for three different angular frequencies, *ω*.

	ω = 0.1 rad/s			ω = 1.0 rad/s			ω = 10. rad/s		
Formulation	G' (Pa)	G'' (Pa)	δ (°)	G' (Pa)	G'' (Pa)	δ (°)	G' (Pa)	G'' (Pa)	δ (°)
Euflexxa	5.17	10.00	62.68	31.90	26.11	39.35	80.84	34.88	24.70
Hyalgan	0.45	0.41	42.65	0.43	0.55	55.58	0.67^	1.49^	160.95^
Hymovis	36.03	19.01	27.82	63.23	17.01	15.07	85.44	15.13	10.65
Monovisc	0.08	0.66	83.26	1.33	5.22	76.26	15.41	28.01	69.61
Supartz	0.35	0.54	57.04	0.52	2.41	78.88	7.13	14.15	81.43
Synvisc	30.93	23.18	36.86	71.68	27.67	21.12	111.51	24.39	12.90

G’, storage modulus; *G”*, loss modulus; *δ*, phase angle

^Indicates that inertial effects of the rheometer head likely dominate this measurement due to low mechanical properties.

### Frictional behavior of HA formulations is not fully described by standard rheology

**Fig 2:**
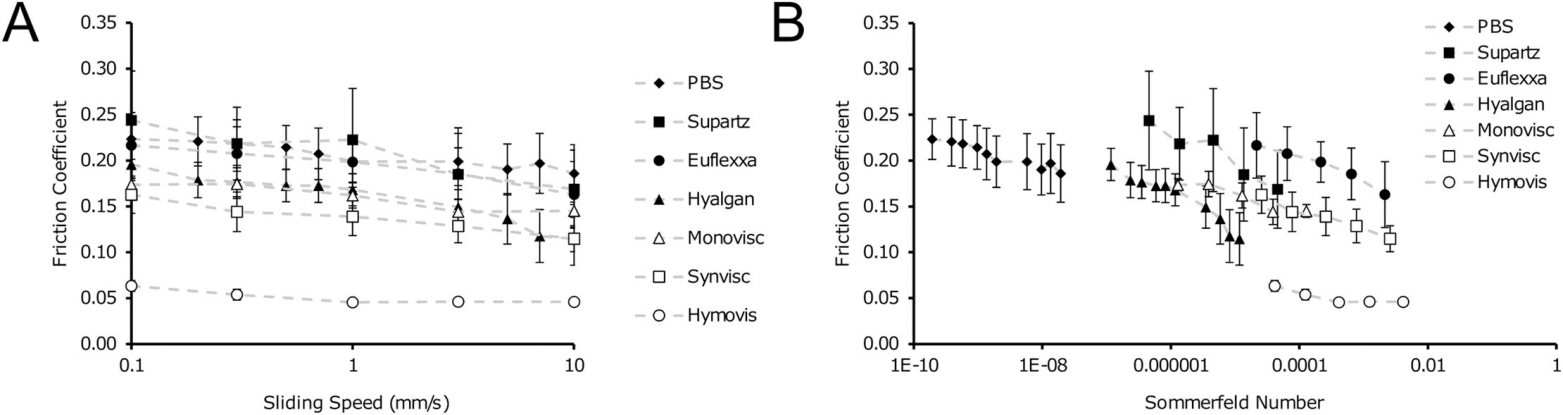
(A) All HA formulations exhibited decreased friction as a function of increased sliding speed. (B) However, presenting friction as a function of the Sommerfeld number (*S*, Eq 2) did not collapse all of the data onto a master friction curve when S was calculated based on η_0_ values obtained in Table **1** (n = 4 samples per group).

Classically, clinical HA injections have been considered mechanical interventions with shock absorbing and lubricating modes of action. To test this hypothesis that HA is effective in lowering the friction coefficient of cartilage, we utilized a previously described tribometer to evaluate the ability of these HA formulations to lower cartilage friction. Briefly, neonatal bovine articular cartilage cylinders were mated against a polished glass counterface, compressed to 25% axial strain, and allowed to equilibrate for 60 minutes while bathed in one of the HA formulations or PBS. The glass slide was then reciprocated through a speed sweep from 0.1 to 10 mm/s, and both normal and shear loads were recorded using a custom biaxial load cell. For each speed, the friction coefficient was recorded as the ratio of the shear load to normal load at the end of the sliding cycle when the friction coefficient reached a steady value. As with viscoelastic properties, the ability of HA formulations to lubricate cartilage varied widely, with friction coefficients ranging from over 0.2 to under 0.05 (**Fig 2**). Additionally, speed-dependence was observed for all lubricants, as would be expected for sliding within highly viscous lubricants. Because of the speed-dependence, we examined friction as a function of the Sommerfeld number, *S*.

$$S=\frac{v\eta d}{N}$$ (Eq 2)

This normalization presents friction as a function of *v* sliding speed, *η* lubricant viscosity, *d* contact width, and *N* normal load. In performing this analysis, the question emerges as to the appropriate viscosity to use for each formulation as they all exhibit viscoelastic, non-Newtonian, shear thinning behavior. We have recently shown that the lubricating behavior of such HA formulations can be described by low shear viscosities [21], thus we incorporated the zero-shear rate viscosities (i.e., *η*_*0*_, **Table 1**) obtained from the Carreau-Yasuda curve fitting. However, upon inspection of the normalized data (**Fig 2B**), it was evident that normalization to this viscosity was not sufficient to collapse the data onto a master curve of friction versus Sommerfeld number. This fact should not be entirely surprising as it is clear that all of the formulations have different shear thinning and elastic properties (**Fig 1 and S2 Fig**). Additionally, we and others have recently reported that HA interacts with the articular surface through bound lubricin, and it is possible that chemical modifications of these HA formulations can alter this interaction [21,39,40].

**Fig 3:**
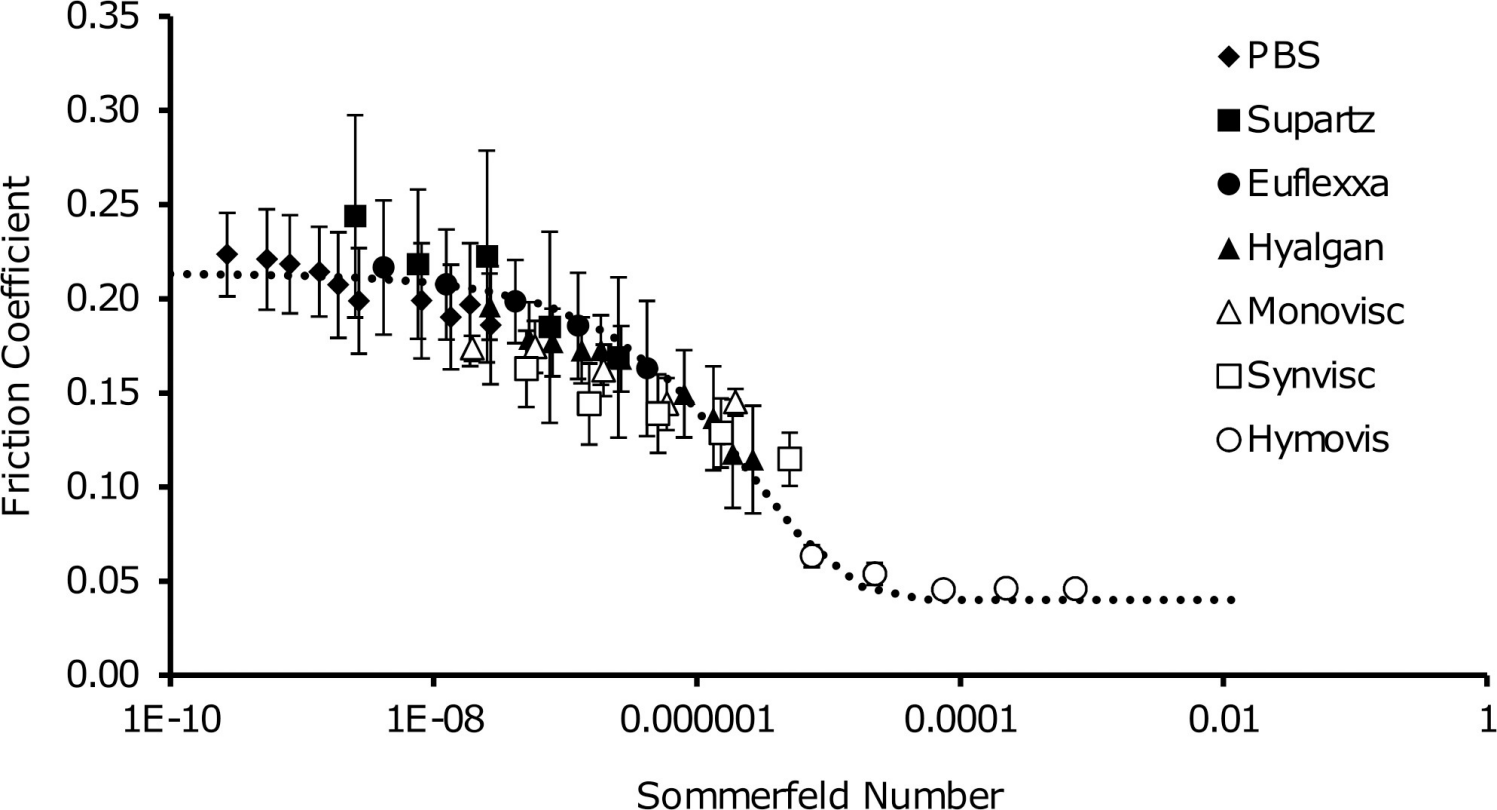
Calculation of an effective lubricating viscosity (η_eff_) collapsed all of the data onto a master friction curve with friction coefficient as a function of the Sommerfeld number (Eq 3).

Thus, to better understand the lubricating behavior of these HA formulations, we allowed *η* to vary when calculating *S* to determine an effective lubricating viscosity, *η*_*eff*_. To calculate this parameter for each lubricant, we compared each friction sweep to a friction curve we previously obtained in the absence of HA. Specifically, we collapsed the data onto an elastoviscous curve obtained for 2 MDa dextran lubricating cartilage (**Fig 3**). This was conducted by minimizing RMS error between the data and the model curve. This curve of friction coefficient μ, as a function of *S* is given by:

$$\mu (S)=\mu_{min}+(\mu_{B}-\mu_{min})e^{-{\left(S/S_{t}\right)}^{d}}$$ (Eq 3)

Where, *μ*_*min*_ is the minimum friction coefficient, *μ*_*B*_ is the boundary friction coefficient, *S*_*t*_ is the Sommerfeld number at the mid-point of the transition from high to low friction, and *d* is a fitting parameter controlling the slope of the transition. The values obtained for 2 MDa dextran are: *μ*_*min*_ = 0.04, *μ*_*B*_ = 0.21, *S*_*t*_ = 2.7·10^−6^, and *d* = 0.62. For each lubricant, the effective viscosity was lower than the measured viscosity, in some cases by orders of magnitude. This difference, which was highly variable between the HA formulations, highlights the fact that chemical modifications of HA formulations can alter the lubricating properties in a manner not fully predicted by the measured viscosity.

### Traditional rheological and viscoelastic characterization does not predict clinical outcome, but friction does

**Fig 4:**
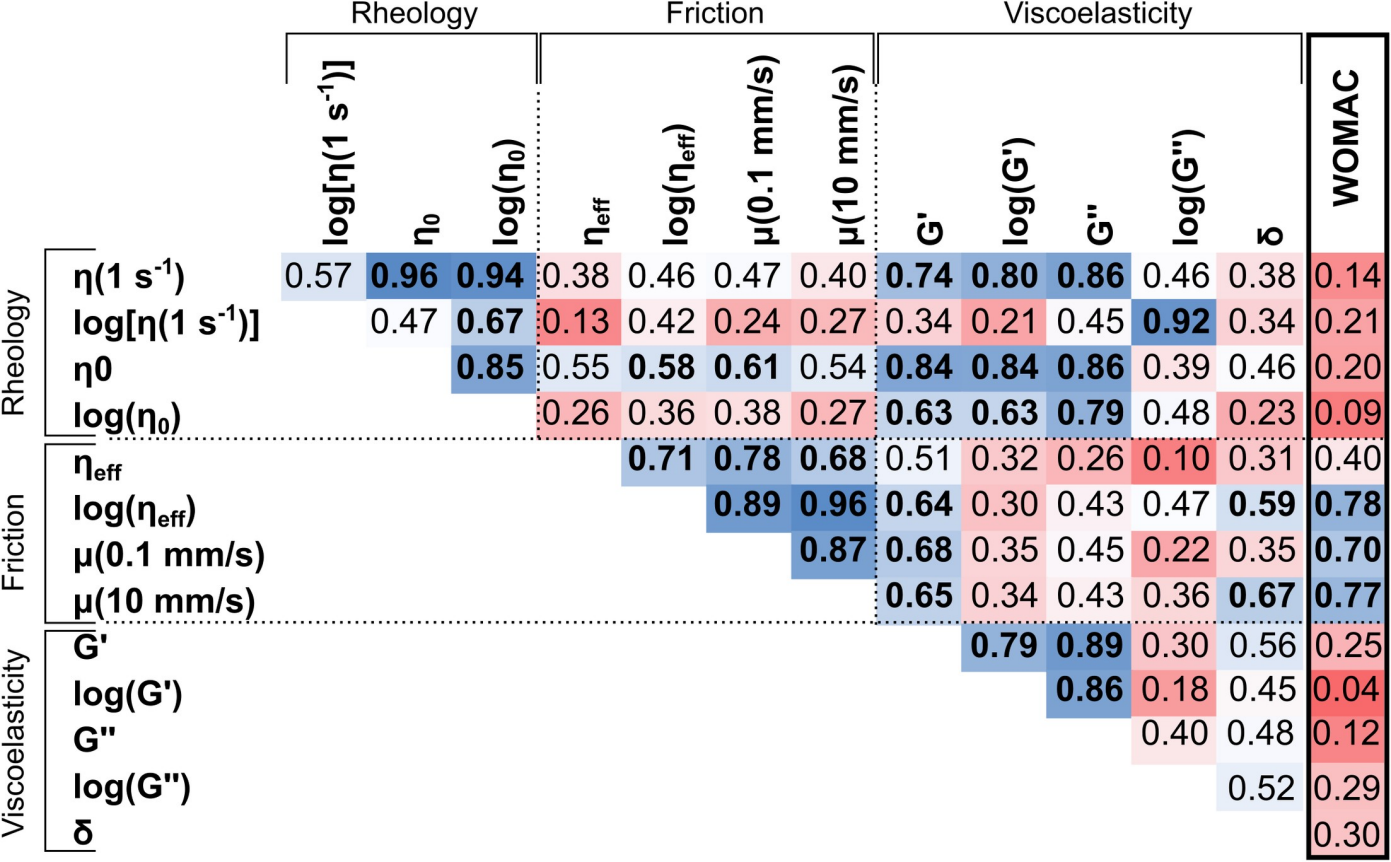
R^2^ values for pair-wise comparisons of rheological properties, friction properties, and changes in WOMAC from baseline. Red indicates poor correlations and blue indicates strong correlations. Bolded values indicate significant correlations with p < 0.05. Of note is the correlation between frictional properties and change in WOMAC.

**Fig 5:**
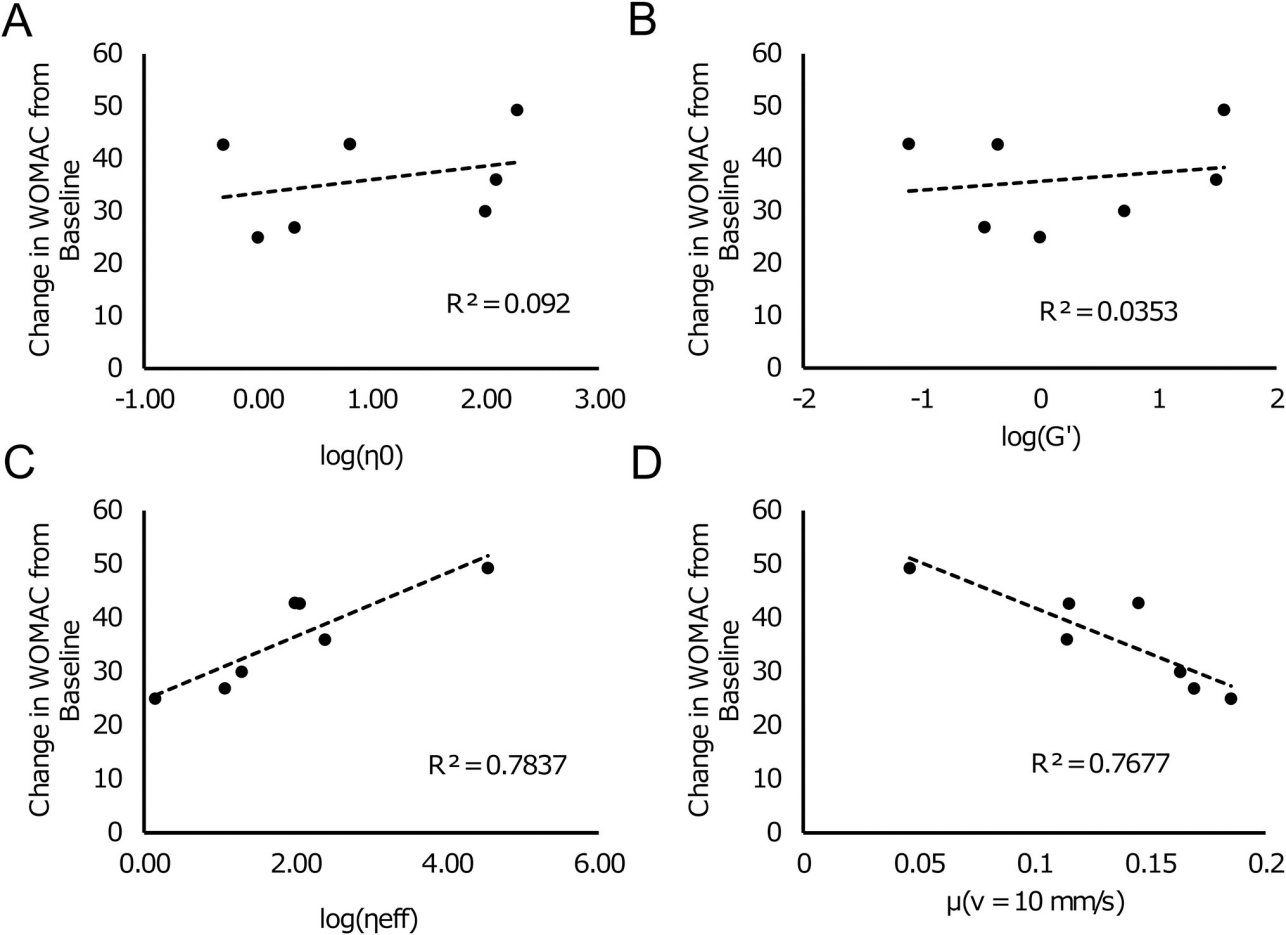
(A,B) Collection of data from all products studied indicated that rheological properties (i.e., η_0_ and G’) were not predictive of mean change in WOMAC score from baseline. (C,D) However, the effective lubricating viscosity (lubricating viscosity (η_eff_) and friction coefficient (μ) provided strong correlations with the clinical data.

We aggregated clinical trial data for each of these HA formulations tested and extracted the change in WOMAC pain score from baseline. First, we inspected correlations between the rheological properties and maximum WOMAC change from baseline. While some rheological properties were predictive of other rheological properties [e.g., R^2^ = 0.92 between η($\dot{\gamma }$ = 0.1) and G”], none of the rheological properties were predictive of clinical outcomes (**Fig 4** and **Fig 5A and 5B**). In fact, the highest correlation coefficient observed was between the phase angle, *δ*, and change in WOMAC (R^2^ = 0.3), but this comparison fell well short of significance (p = 0.2). Interestingly, the rheological properties of the formulations were not predictive of the frictional behavior either. The strongest correlation; however, was between the zero shear viscosity and the friction coefficient at 0.1 mm/s (R^2^ = 0.61, p = 0.04, **Fig 4**). Additionally, in stark contrast to the rheological data, the measured friction coefficients and the effective viscosity calculated from the frictional analysis were much more predictive of the change in WOMAC scores (**Fig 4**, **Fig 5C and 5D**). In fact, low speed friction [μ(v = 0.1 mm/s)], high speed friction [μ(v = 10 mm/s)], and the effective viscosity (η_eff_) all provided significant correlations with the aggregated clinical outcome data (R^2^ = 0.70, p = 0.019; R^2^ = 0.77, p = 0.009; and R^2^ = 0.78, p = 0.008, respectively).

## Discussion

Here, we assessed both the rheological and tribological properties of clinically approved hyaluronic acid viscosupplements and found that data obtained from cartilage friction measurements are significantly more predictive of published clinical outcomes than either rheological or viscoelastic properties as measured by traditional means. Indeed, despite the colloquial use of the term ‘viscosupplementation’, we found little evidence that these products can be judged in a pre-clinical context solely by their rheological properties measured with standard techniques.

A major question remains as to connection between frictional properties of these formulations and the maximum reduction of pain reported by the WOMAC scores (**Fig 4** and **Fig 5C and 5D**). There is currently growing evidence that chondrocytes are susceptible to dysfunction as a direct result of altered friction levels within a joint [44–46]. Additionally, there is a strong association between cartilage friction and wear of the articular surface [47,48]. While these factors may provide possible clues to the relationships found in this study, there is a host of possible explanations for this correlation.

One particularly interesting finding of this study revealed that the widely varying viscosities of these formulations did not predict the frictional properties. In fact, the measured viscosities, η_0_, varied by orders of magnitude from effective viscosities, η_eff_, in an unpredictable manner. This result may be linked to difficulties in measuring rheological properties in a robust and physiologically relevant manner. For highly viscous polymeric solutions such as the ones studied here, factors such as wall slip and interfacial effects may lead to this disconnect [38,49]. Thus, standard techniques that do not account for such interfacial effects may not accurately measure mechanical properties. It should be noted that for other biomolecules such as mucin [50], adsorption to a surface causes an increased local viscosity that enhances lubricating properties, and we have recently shown that a similar mechanism can occur for HA [21]. In fact, tuning the affinity of HA to the cartilage surface can drastically alter the lubricating response [51]. With this in mind, it is currently unclear how HA-stainless steel interfaces in rheological configurations mimic the physiologically relevant cartilage-HA interface. Further, we and others have recently reported that HA interacts with molecules such as lubricin bound to the articular surface [21,39,40]. This interaction with lubricin is not specific to HA and can be replicated by another viscous polysaccharide, dextran. It is hypothesized that facilitating the aggregation of HA at the articular surface allows viscous surface layers to develop, which in turn facilitate lubrication through a mechanism called viscous boundary lubrication [50]. It is possible that the molecular weight variations and the chemical modifications of the HA formulations in the present study alter such interactions with the articular surface that can either promote or inhibit effective lubrication leading to the disparities observed.

While this study revealed a strong connection between lubricating properties and clinical data, there are several limitations that must be addressed. This study utilized healthy, neonatal bovine cartilage to mitigate sample to sample variations that could occur during testing. We have previously shown, however, that both injury and degeneration alter the lubrication of cartilage by HA [52,53], thus the frictional properties reported may not fully describe the lubricating effect of the HA formulations on clinically relevant tissue, and the reported friction coefficients may not fully recapitulate the in vivo situation. While HA and other synovial fluid molecules lower the cartilage friction coefficient, the structure of cartilage is also crucial to the low friction surfaces as the low permeability of the tissue allows substantial interstitial fluid pressurization [54,55]. Additionally, the rheological techniques used do not necessarily represent the state of the art, but represent standard techniques that may not capture the complex mechanical properties of these HA solutions. These techniques may not fully convey rheological phenomenon that occur at higher or lower strains, strain rates and oscillation frequencies in addition to interfacial effects that likely contribute to the frictional response. Further, the clinical trial data was not aggregated from studies conducted in an identical manner. Aspects including doses, timing of administration, timing of maximum efficacy, and study inclusion/exclusion criteria all varied between the clinical trials. It is also noted that the studies aggregated for this analysis are not exhaustive of the clinical data, but represent a subset of studies where both the clinical data were available and the lubricant was available for in vitro testing. Additionally, it should be noted that despite the strong correlations, causation is not necessarily implied. Other factors that can trend with lubricating efficacy may also play a vital role. For example, altering the chemical structure to promote effective lubrication can have a parallel effect of altering the residence time of these molecules. Thus promoting aggregation of HA at the articular surface could promote lubrication and enhance residence time simultaneously. Despite these stated limitations, this study revealed that rheological and viscoelastic properties are less predictive of clinical efficacy compared to lubricating ability.

## Materials and methods

### Rheological testing

To determine the role of viscosity, a commercial rheometer (TA Instruments DHR3 Rheometer, New Castle DE) was used to measure the shear rate-dependent viscosity of the lubricant baths. For the HA-based lubricants, a 40 mm diameter cone-plate set up with a 2° angle and 50 μm truncation was used in a shear rate sweep of $\dot{\gamma }={0.1\;to\;1000\;s}^{-1}$ to determine dynamic viscosities based on standard protocols from the manufacturer built into the Trios software package. To determine the pseudoplastic properties of the lubricating solutions, the shear rate ($\dot{\gamma }$) dependent dynamic viscosity (*η*) was fit to a Carreau-Yasuda model given by Eq 1. The parameters were determined by minimizing the root-mean-square error between the data and the model fit using a custom Excel template. Goodness of fit was reported for each curve based on the coefficient of variation of the RMS error. Additionally, this rheometer configuration (40 mm cone-plate) was utilized to determine viscoelastic properties of the solutions (i.e., storage and loss moduli, and phase angle). Preliminary evidence suggested that the linear regime for HA and modified HA solutions extends past 10% strain [56,57]. To capture this behavior, these analyses were conducted using 3% oscillatory shear strain with an angular frequency sweep from ω = 0.1 to 100 rad/sec based on protocols in the Trios software. Data are reported for a single replicate from the same lubricant formulation batch used in the tribological testing described below.

### Tribological testing

Tribological testing was conducted as described recently [21,52,53]. Friction coefficients of cartilage-against-glass were measured on a custom tribometer. Cartilage samples were extracted from the patellofemoral groove of neonatal (1–3 day old) bovine stifles. These cartilage samples were extracted using a 6 mm diameter biopsy punch and sized to 2 mm thick cylinders. Cartilage was mated against a polished glass flat counterface while bathed in a lubricant bath in a tilt-pad bearing configuration [58]. Friction coefficients were measured in a stationary contact area configuration. That is, cartilage was compressed against a flat surface and reciprocated in a manner that mitigates the effects of interstitial fluid pressurization on friction coefficient measurements that can arise from active deformation of the cartilage matrix. Prior to friction coefficient measurements, samples were compressed to 25% axial strain and allowed to depressurize over the course of 1 hour resulting in equilibrium normal loads on the order of 2.5 N. Following normal force equilibrium, the glass counterface was reciprocated at predetermined speeds ranging from 0.1 to 10 mm/s, and friction coefficients were calculated as the ratio of shear load to normal load measured by a biaxial load cell. Coefficients were calculated at the end of sliding when friction had reached an equilibrium value to mitigate effects of the static friction coefficient and inertial effects that are present at the beginning of sliding at elevated speeds. To account for any misalignments, the friction coefficient was averaged for both the forward and reverse sliding directions.

### Lubricant formulations and cartilage surfaces

Lubricants used in this study were phosphate buffered saline (PBS; Corning, Manassas VA). Sodium hyaluronate with 500–730 kDa molecular weight obtained from *Streptococcus Equi* fermentation and formulated to a final solution of 10 mg/mL in PBS (Hyalgan, Fidia Farmaceutici, Padua Italy) was used as the HA solution. Hymovis (Fidia Farmaceutici, Padua Italy), which is based on HYADD4, a hydrophobic partial hexadecyl derivative of HA with a degree of substitution ~ 2% mol/mol with respect to the polysaccharide repeating unit, provided a lubricant bath with increased viscosity at a concentration of 8 mg/mL in PBS. Additionally, other commercially available viscosupplements were tested at their clinically relevant concentrations. These solutions were Supartz (trademark of Seikugaku Corporation), Monovisc (trademark of Anika Therapeutics), Synvisc (trademark of Genzyme Corporation), and Euflexxa (trademark of Ferring BV) (Gifts from Drs. Scott Rodeo and John Kennedy, Hospital for Special Surgery).

### Comparison to clinical trial data

To assess the extent to which data from friction studies and rheological characterization correlated with clinical outcomes, data was collected from published clinical trials [32,59–64]. For all data sets, the parameter chosen to represent clinical efficacy was the maximum percentage improvement in WOMAC score compared to baseline, regardless of the time point at which such maximal improvement occurred. Because placebo effects due to saline injections are often quite high, the comparison of clinical efficacy to tribological and rheological studies also included data on saline injection, using measured parameters on the properties of PBS to enable inclusion of such data in correlation analyses [9].

### Statistical analysis

Rheological data from flow sweep experiments were fit to a Carreau-Yasuda model to determine the zero shear rate viscosity (Eq 1). These calculations were conducted in a custom excel file that determined the five fitting parameters through root-mean-squared error minimization. Additionally, the coefficient of variation of the RMS error were calculated and reported. Friction data were plotted as a function of *S* (Eq 2) and fit to a friction transition curve (Eq 3) by minimizing RMS error. Correlations between measured parameters and clinical trial data were conducted through linear regression and R^2^ values were reported in **Fig 4** for each comparison. Significance was determined using a Pearson correlation coefficient.

## Supporting information

S1 Fig Example flow sweep viscosity data along with the associated Carreau-Yasuda fit. (TIF)

S2 Fig Oscillatory shear data for all HA formulations. A cross-over frequency was not evident within the operating conditions for all of the formulations.
(TIF)

## Abbreviations

A: Carreau-Yasuda fitting parameter
d: friction fitting parameter
G': storage modulus
G'': loss modulus
HA: hyaluronic acid
N: normal load
n: Carreau-Yasuda fitting parameter
S: Sommerfeld number
S_t_: Sommerfeld transition number
v: sliding speed
WOMAC: Western Ontario and McMaster Universities Osteoarthritis Index
$\dot{\gamma }$: shear rate
δ: phase angle
η: viscosity
η_0_: zero shear rate viscosity
η_∞_: infinite shear rate viscosity
η_eff_: effective lubricating viscosity
λ: Carreau-Yasuda fitting parameter
μ: friction coefficient
μ_B_: boundary friction coefficient
μ_min_: minimum friction coefficient
ω: angular frequency

## References

[1] Namiki O, Toyoshima H, Morisaki N. Therapeutic effect of intra-articular injection of high molecular weight hyaluronic acid on osteoarthritis of the knee. Int J Clin Pharmacol Ther Toxicol. 1982;20: 501–7. Available: http://www.ncbi.nlm.nih.gov/pubmed/7174151 7174151

[2] Dixon ASJ, Jacoby RK, Berry H, Hamilton EBD. Clinical trial of intra-articular injection of sodium hyaluronate in patients with osteoarthritis of the knee. Curr Med Res Opin. Taylor & Francis; 1988;11: 205–213. 10.1185/03007998809114237 3063436

[3] Peyron JG. A new approach to the treatment of osteoarthritis: viscosupplementation. Osteoarthr Cartil. 1993;1: 85–7. 10.1016/S1063-4584(05)80022-6 8886083

[4] Jevsevar DS. Treatment of Osteoarthritis of the Knee: Evidence-Based Guideline, 2nd Edition. J Am Acad Orthop Surg. 2013;21: 571–576. 10.5435/JAAOS-21-09-571 23996988

[5] Henrotin Y, Raman R, Richette P, Bard H, Jerosch J, Conrozier T, et al Consensus statement on viscosupplementation with hyaluronic acid for the management of osteoarthritis. Semin Arthritis Rheum. Elsevier; 2015;45: 140–149. 10.1016/j.semarthrit.2015.04.011 26094903

[6] Trojian TH, Concoff AL, Joy SM, Hatzenbuehler JR, Saulsberry WJ, Coleman CI. AMSSM scientific statement concerning viscosupplementation injections for knee osteoarthritis: importance for individual patient outcomes. Br J Sports Med. BMJ Publishing Group Ltd and British Association of Sport and Exercise Medicine; 2016;50: 84–92. 10.1136/bjsports-2015-095683 26729890

[7] Hochberg MC, Altman RD, April KT, Benkhalti M, Guyatt G, McGowan J, et al American College of Rheumatology 2012 recommendations for the use of nonpharmacologic and pharmacologic therapies in osteoarthritis of the hand, hip, and knee. Arthritis Care Res (Hoboken). 2012;64: 465–74. Available: http://www.ncbi.nlm.nih.gov/pubmed/22563589 22563589 10.1002/acr.21596

[8] Bannuru RR, Vaysbrot EE, McIntyre LF. Did the American Academy of Orthopaedic Surgeons Osteoarthritis Guidelines Miss the Mark? Arthrosc J Arthrosc Relat Surg. W.B. Saunders; 2014;30: 86–89. 10.1016/J.ARTHRO.2013.10.007 24384274

[9] Bannuru RR, Schmid CH, Kent DM, Vaysbrot EE, Wong JB, McAlindon TE. Comparative Effectiveness of Pharmacologic Interventions for Knee Osteoarthritis. Ann Intern Med. 2015;162: 46 10.7326/M14-1231 25560713

[10] Viscosupplementation Market to Hit $2.6 Billion by 2021 [Internet]. [cited 28 Aug 2018]. Available: https://www.prnewswire.com/news-releases/viscosupplementation-market-to-hit-26-billion-by-2021-528457891.html

[11] Knudson CB. Hyaluronan receptor-directed assembly of chondrocyte pericellular matrix. J Cell Biol. The Rockefeller University Press; 1993;120: 825–34. Available: http://www.ncbi.nlm.nih.gov/pubmed/7678838 7678838 10.1083/jcb.120.3.825 PMC2119530

[12] Waddell DD, Kolomytkin O V, Dunn S, Marino AA. Hyaluronan suppresses IL-1beta-induced metalloproteinase activity from synovial tissue. Clin Orthop Relat Res. 2007;465: 241–8. 10.1097/BLO.0b013e31815873f9 18090474

[13] Yatabe T, Mochizuki S, Takizawa M, Chijiiwa M, Okada A, Kimura T, et al Hyaluronan inhibits expression of ADAMTS4 (aggrecanase-1) in human osteoarthritic chondrocytes. Ann Rheum Dis. BMJ Publishing Group; 2009;68: 1051–8. 10.1136/ard.2007.086884 18662930 PMC2674548

[14] Creamer P, Sharif M, George E, Meadows K, Cushnaghan J, Shinmei M, et al Intra-articular hyaluronic acid in osteoarthritis of the knee: an investigation into mechanisms of action. Osteoarthr Cartil. 1994;2: 133–40. Available: http://www.ncbi.nlm.nih.gov/pubmed/11548229 11548229 10.1016/s1063-4584(05)80063-9

[15] Li J, Gorski DJ, Anemaet W, Velasco J, Takeuchi J, Sandy JD, et al Hyaluronan injection in murine osteoarthritis prevents TGFbeta 1-induced synovial neovascularization and fibrosis and maintains articular cartilage integrity by a CD44-dependent mechanism. Arthritis Res Ther. BioMed Central; 2012;14: R151 10.1186/ar3887 22721434 PMC3446537

[16] Kobayashi K, Matsuzaka S, Yoshida Y, Miyauchi S, Wada Y, Moriya H. The effects of intraarticularly injected sodium hyaluronate on levels of intact aggrecan and nitric oxide in the joint fluid of patients with knee osteoarthritis. Osteoarthr Cartil. 2004;12: 536–542. 10.1016/j.joca.2004.03.005 15219568

[17] Swann DA, Radin EL, Nazimiec M, Weisser PA, Curran N, Lewinnek G. Role of hyaluronic acid in joint lubrication. Ann Rheum Dis. 1974;33: 318–26. Available: http://www.pubmedcentral.nih.gov/articlerender.fcgi?artid=1006265&tool=pmcentrez&rendertype=abstract 10.1136/ard.33.4.318 4415649 PMC1006265

[18] Mori S, Naito M, Moriyama S. Highly viscous sodium hyaluronate and joint lubrication. Int Orthop. Springer; 2002;26: 116–21. 10.1007/S00264-002-0330-Z 12078873 PMC3620869

[19] Murakami T, Higaki H, Sawae Y, Ohtsuki N, Moriyama S, Nakanishi Y. Adaptive multimode lubrication in natural synovial joints and artificial joints. Proc Inst Mech Eng Part H J Eng Med. 1998;212: 23–35. 10.1243/0954411981533791 9529934

[20] Schmidt TA, Gastelum NS, Nguyen QT, Schumacher BL, Sah RL. Boundary lubrication of articular cartilage: role of synovial fluid constituents. Arthritis Rheum. 2007;56: 882–91. 10.1002/art.22446 17328061

[21] Bonnevie ED, Galesso D, Secchieri C, Cohen I, Bonassar LJ. Elastoviscous Transitions of Articular Cartilage Reveal a Mechanism of Synergy between Lubricin and Hyaluronic Acid. PLoS One. Public Library of Science; 2015;10: e0143415 10.1371/journal.pone.0143415 26599797 PMC4658013

[22] Mendichi Raniero *,†, Ladislav Šoltés ‡ and, Schieroni AG. Evaluation of Radius of Gyration and Intrinsic Viscosity Molar Mass Dependence and Stiffness of Hyaluronan. American Chemical Society; 2003; 10.1021/BM0342178 14606912

[23] Antonacci JM, Schmidt TA, Serventi LA, Cai MZ, Shu YL, Schumacher BL, et al Effects of equine joint injury on boundary lubrication of articular cartilage by synovial fluid: role of hyaluronan. Arthritis Rheum. NIH Public Access; 2012;64: 2917–26. 10.1002/art.34520 22605527 PMC3424370

[24] al-Assaf S, Meadows J, Phillips GO, Williams PA. The application of shear and extensional viscosity measurements to assess the potential of hylan in viscosupplementation. Biorheology. 33: 319–32. Available: http://www.ncbi.nlm.nih.gov/pubmed/8977658 8977658 10.1016/0006-355x(96)00025-x

[25] Kikuchi T, Yamada H, Shimmei M. Effect of high molecular weight hyaluronan on cartilage degeneration in a rabbit model of osteoarthritis. Osteoarthr Cartil. 1996;4: 99–110. Available: http://www.ncbi.nlm.nih.gov/pubmed/8806112 8806112 10.1016/s1063-4584(05)80319-x

[26] Maneiro E, de Andres MC, Fernández-Sueiro JL, Galdo F, Blanco FJ. The biological action of hyaluronan on human osteoartritic articular chondrocytes: the importance of molecular weight. Clin Exp Rheumatol. 22: 307–12. Available: http://www.ncbi.nlm.nih.gov/pubmed/15144124 15144124

[27] Wobig M, Bach G, Beks P, Dickhut A, Runzheimer J, Schwieger G, et al The role of elastoviscosity in the efficacy of viscosupplementation for osteoarthritis of the knee: a comparison of hylan G-F 20 and a lower-molecular-weight hyaluronan. Clin Ther. 1999;21: 1549–62. Available: http://www.ncbi.nlm.nih.gov/pubmed/10509850 10509850 10.1016/s0149-2918(00)80010-7

[28] Bayramoğlu M, Karataş M, Çetin N, Akman N, Sözay S, Dilek A. Comparison of two different viscosupplements in knee osteoarthritis—a pilot study. Clin Rheumatol. 2003;22: 118–122. 10.1007/s10067-002-0691-0 12740676

[29] Kawano T, Miura H, Mawatari T, Moro-Oka T, Nakanishi Y, Higaki H, et al Mechanical effects of the intraarticular administration of high molecular weight hyaluronic acid plus phospholipid on synovial joint lubrication and prevention of articular cartilage degeneration in experimental osteoarthritis. Arthritis Rheum. 2003;48: 1923–1929. 10.1002/art.11172 12847686

[30] Tıkız C, Ünlü Z, Şener A, Efe M, Tüzün Ç. Comparison of the efficacy of lower and higher molecular weight viscosupplementation in the treatment of hip osteoarthritis. Clin Rheumatol. 2005;24: 244–250. 10.1007/s10067-004-1013-5 15647968

[31] Kotevoglu N, Iyıbozkurt PC, Hız O, Toktas H, Kuran B. A prospective randomised controlled clinical trial comparing the efficacy of different molecular weight hyaluronan solutions in the treatment of knee osteoarthritis. Rheumatol Int. 2006;26: 325–330. 10.1007/s00296-005-0611-0 15959784

[32] Kirchner M, Marshall D. A double-blind randomized controlled trial comparing alternate forms of high molecular weight hyaluronan for the treatment of osteoarthritis of the knee. Osteoarthr Cartil. 2006;14: 154–162. 10.1016/j.joca.2005.09.003 16242361

[33] Lee P, Kim Y, Lim Y, Lee C, Sim W, Ha C, et al Comparison between High and Low Molecular Weight Hyaluronates in Knee Osteoarthritis Patients: Open-label, Randomized, Multicentre Clinical Trial. J Int Med Res. 2006;34: 77–87. 10.1177/147323000603400110 16604827

[34] Maheu E, Zaim M, Appelboom T, Jeka S, Trc T, Berenbaum F, et al Comparative efficacy and safety of two different molecular weight (MW) hyaluronans F60027 and Hylan G-F20 in symptomatic osteoarthritis of the knee (KOA). Results of a non inferiority, prospective, randomized, controlled trial. Clin Exp Rheumatol. 29: 527–35. Available: http://www.ncbi.nlm.nih.gov/pubmed/21722501 21722501

[35] Pavelka K, Uebelhart D. Efficacy evaluation of highly purified intra-articular hyaluronic acid (Sinovial®) vs hylan G-F20 (Synvisc®) in the treatment of symptomatic knee osteoarthritis. A double-blind, controlled, randomized, parallel-group non-inferiority study. Osteoarthr Cartil. 2011;19: 1294–1300. 10.1016/j.joca.2011.07.016 21875678

[36] Vitanzo PC, Sennett BJ. Hyaluronans: is clinical effectiveness dependent on molecular weight? Am J Orthop (Belle Mead NJ). 2006;35: 421–8. Available: http://www.ncbi.nlm.nih.gov/pubmed/17036778 17036778

[37] Ghosh P, Guidolin D. Potential mechanism of action of intra-articular hyaluronan therapy in osteoarthritis: are the effects molecular weight dependent? Semin Arthritis Rheum. 2002;32: 10–37. Available: http://www.ncbi.nlm.nih.gov/pubmed/12219318 12219318 10.1053/sarh.2002.33720

[38] Zhang Z, Barman S, Christopher GF. The role of protein content on the steady and oscillatory shear rheology of model synovial fluids. Soft Matter. The Royal Society of Chemistry; 2014;10: 5965–5973. 10.1039/c4sm00716f 24989639

[39] Das S, Banquy X, Zappone B, Greene GW, Jay GD, Israelachvili JN. Synergistic interactions between grafted hyaluronic acid and lubricin provide enhanced wear protection and lubrication. Biomacromolecules. American Chemical Society; 2013;14: 1669–77. 10.1021/bm400327a 23560944

[40] Majd SE, Kuijer R, Köwitsch A, Groth T, Schmidt TA, Sharma PK. Both hyaluronan and collagen type II keep proteoglycan 4 (lubricin) at the cartilage surface in a condition that provides low friction during boundary lubrication. Langmuir. American Chemical Society; 2014;30: 14566–72. 10.1021/la504345c 25409034

[41] Stewart T, Jin ZM, Fisher J. Friction of composite cushion bearings for total knee joint replacements under adverse lubrication conditions. Proc Inst Mech Eng Part H J Eng Med. SAGE PublicationsSage UK: London, England; 1997;211: 451–465. 10.1243/0954411981534574 9509883

[42] Shi L, Sikavitsas VI, Striolo A. Experimental Friction Coefficients for Bovine Cartilage Measured with a Pin-on-Disk Tribometer: Testing Configuration and Lubricant Effects. Ann Biomed Eng. 2011;39: 132–146. 10.1007/s10439-010-0167-3 20872073

[43] Dunn AC, Sawyer WG, Angelini TE. Gemini Interfaces in Aqueous Lubrication with Hydrogels. Tribol Lett. 2014;54: 59–66. 10.1007/s11249-014-0308-1

[44] Waller KA, Zhang LX, Jay GD. Friction-Induced Mitochondrial Dysregulation Contributes to Joint Deterioration in Prg4 Knockout Mice. Int J Mol Sci. 2017;18: 1252 10.3390/ijms18061252 28604608 PMC5486075

[45] Waller KA, Zhang LX, Elsaid KA, Fleming BC, Warman ML, Jay GD. Role of lubricin and boundary lubrication in the prevention of chondrocyte apoptosis. Proc Natl Acad Sci U S A. 2013;110: 5852–7. 10.1073/pnas.1219289110 23530215 PMC3625316

[46] Bonnevie ED, Delco ML, Bartell LR, Jasty N, Cohen I, Fortier LA, et al Microscale frictional strains determine chondrocyte fate in loaded cartilage. J Biomech. Elsevier; 2018; 10.1016/J.JBIOMECH.2018.04.020 29729853 PMC6367731

[47] Jay GD, Torres JR, Rhee DK, Helminen HJ, Hytinnen MM, Cha C-J, et al Association between friction and wear in diarthrodial joints lacking lubricin. Arthritis Rheum. 2007;56: 3662–9. 10.1002/art.22974 17968947 PMC2688668

[48] Oungoulian SR, Durney KM, Jones BK, Ahmad CS, Hung CT, Ateshian GA. Wear and damage of articular cartilage with friction against orthopedic implant materials. J Biomech. 2015;48: 1957–1964. 10.1016/j.jbiomech.2015.04.008 25912663 PMC4492870

[49] Sabzevari SM, Cohen I, Wood-Adams PM. Wall Slip of Bidisperse Linear Polymer Melts. Macromolecules. American Chemical Society; 2014;47: 3154–3160. 10.1021/ma500451g

[50] Yakubov GE, McColl J, Bongaerts JHH, Ramsden JJ. Viscous boundary lubrication of hydrophobic surfaces by mucin. Langmuir. American Chemical Society; 2009;25: 2313–21. 10.1021/la8018666 19146419

[51] Singh A, Corvelli M, Unterman SA, Wepasnick KA, McDonnell P, Elisseeff JH. Enhanced lubrication on tissue and biomaterial surfaces through peptide-mediated binding of hyaluronic acid. Nat Mater. 2014;13: 988–995. 10.1038/nmat4048 25087069 PMC6317357

[52] Bonnevie ED, Galesso D, Secchieri C, Bonassar LJ. Degradation alters the lubrication of articular cartilage by high viscosity, hyaluronic acid-based lubricants. J Orthop Res. Wiley-Blackwell; 2017; 10.1002/jor.23782 29068482

[53] Bonnevie ED, Delco ML, Galesso D, Secchieri C, Fortier LA, Bonassar LJ. Sub-critical impact inhibits the lubricating mechanisms of articular cartilage. J Biomech. 2017;53 10.1016/j.jbiomech.2016.12.034 28069163

[54] Krishnan R, Kopacz M, Ateshian GA. Experimental verification of the role of interstitial fluid pressurization in cartilage lubrication. J Orthop Res. 2004;22: 565–70. 10.1016/j.orthres.2003.07.002 15099636 PMC2842190

[55] Ateshian GA. The role of interstitial fluid pressurization in articular cartilage lubrication. J Biomech. 2009;42: 1163–76. 10.1016/j.jbiomech.2009.04.040 19464689 PMC2758165

[56] Hyun K, Kim SH, Ahn KH, Lee SJ. Large amplitude oscillatory shear as a way to classify the complex fluids. J Nonnewton Fluid Mech. 2002;107: 51–65. 10.1016/S0377-0257(02)00141-6

[57] Finelli I, Chiessi E, Galesso D, Renier D, Paradossi G. A new viscosupplement based on partially hydrophobic hyaluronic acid: A comparative study. Biorheology. 2011;48: 263–275. Available: http://iospress.metapress.com/content/k6356u4785148g0q/ 10.3233/BIR-2011-0596 22433568

[58] Gleghorn JP, Bonassar LJ. Lubrication mode analysis of articular cartilage using Stribeck surfaces. J Biomech. 2008;41: 1910–8. 10.1016/j.jbiomech.2008.03.043 18502429

[59] ClinicalTrials.Gov. Study of Safety and Efficacy of 6 mL Synvisc-One (Hylan G-F 20) in Indian Patients With Symptomatic Osteoarthritis of Knee(s) After Initial and Repeat Treatment (NCT02389452) [Internet]. 2017 [cited 3 Oct 2018]. Available: https://clinicaltrials.gov/ct2/show/NCT02389452

[60] ClinicalTrials.Gov. HymovisTM Versus Placebo in Knee Osteoarthritis (NCT01372475) [Internet]. 2014 [cited 3 Oct 2018]. Available: https://clinicaltrials.gov/ct2/show/NCT01372475

[61] Strand V, Baraf HSB, Lavin PT, Lim S, Hosokawa H. A multicenter, randomized controlled trial comparing a single intra-articular injection of Gel-200, a new cross-linked formulation of hyaluronic acid, to phosphate buffered saline for treatment of osteoarthritis of the knee. Osteoarthr Cartil. W.B. Saunders; 2012;20: 350–356. 10.1016/j.joca.2012.01.013 22342928

[62] Day R, Brooks P, Conaghan PG, Petersen M, Multicenter Trial Group MT. A double blind, randomized, multicenter, parallel group study of the effectiveness and tolerance of intraarticular hyaluronan in osteoarthritis of the knee. J Rheumatol. The Journal of Rheumatology; 2004;31: 775–82. Available: http://www.ncbi.nlm.nih.gov/pubmed/15088306 15088306

[63] Altman RD, Moskowitz R. Intraarticular sodium hyaluronate (Hyalgan) in the treatment of patients with osteoarthritis of the knee: a randomized clinical trial. Hyalgan Study Group. J Rheumatol. 1998;25: 2203–12. Available: http://www.ncbi.nlm.nih.gov/pubmed/9818665 9818665

[64] Fidia Parma USA. Hymovis [Package Insert]. Floram Park, NJ; 2015.

