# Supplementary figures and images for: Frictional characterization of injectable hyaluronic acids is more predictive of clinical outcomes than traditional rheological or viscoelastic characterization
Source: PLoS One. 2019 May 10;14(5):e0216702. doi: 10.1371/journal.pone.0216702 (PMC6510437; doi:10.1371/journal.pone.0216702)

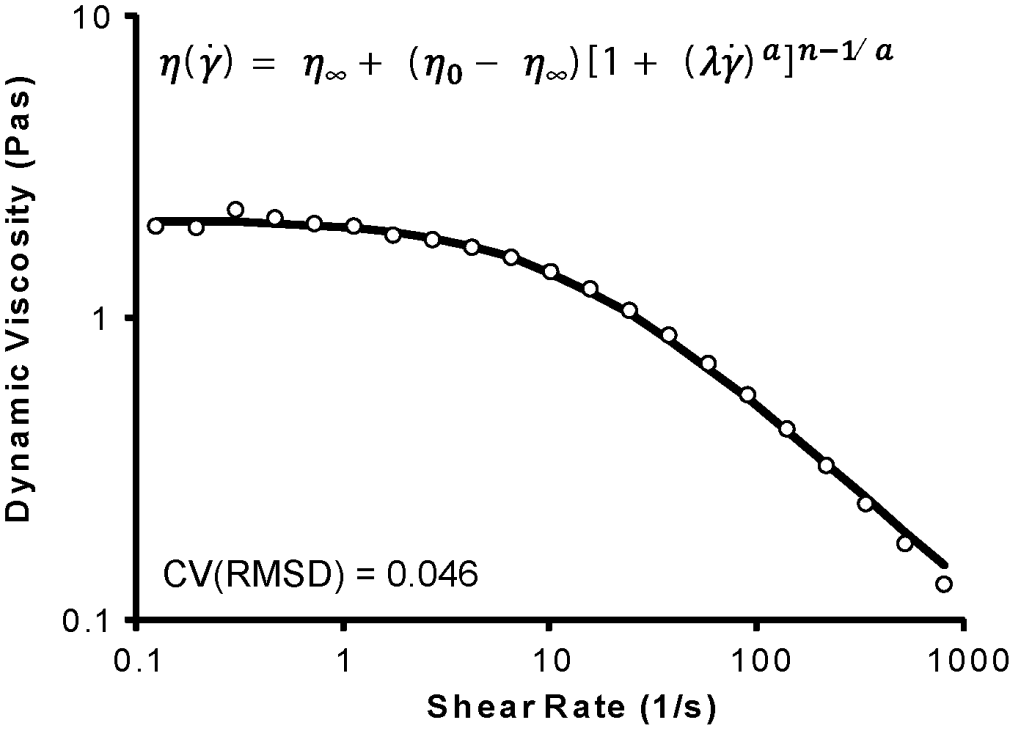

Supplement: S1 Fig — (TIF) [file pone.0216702.s001.tif]

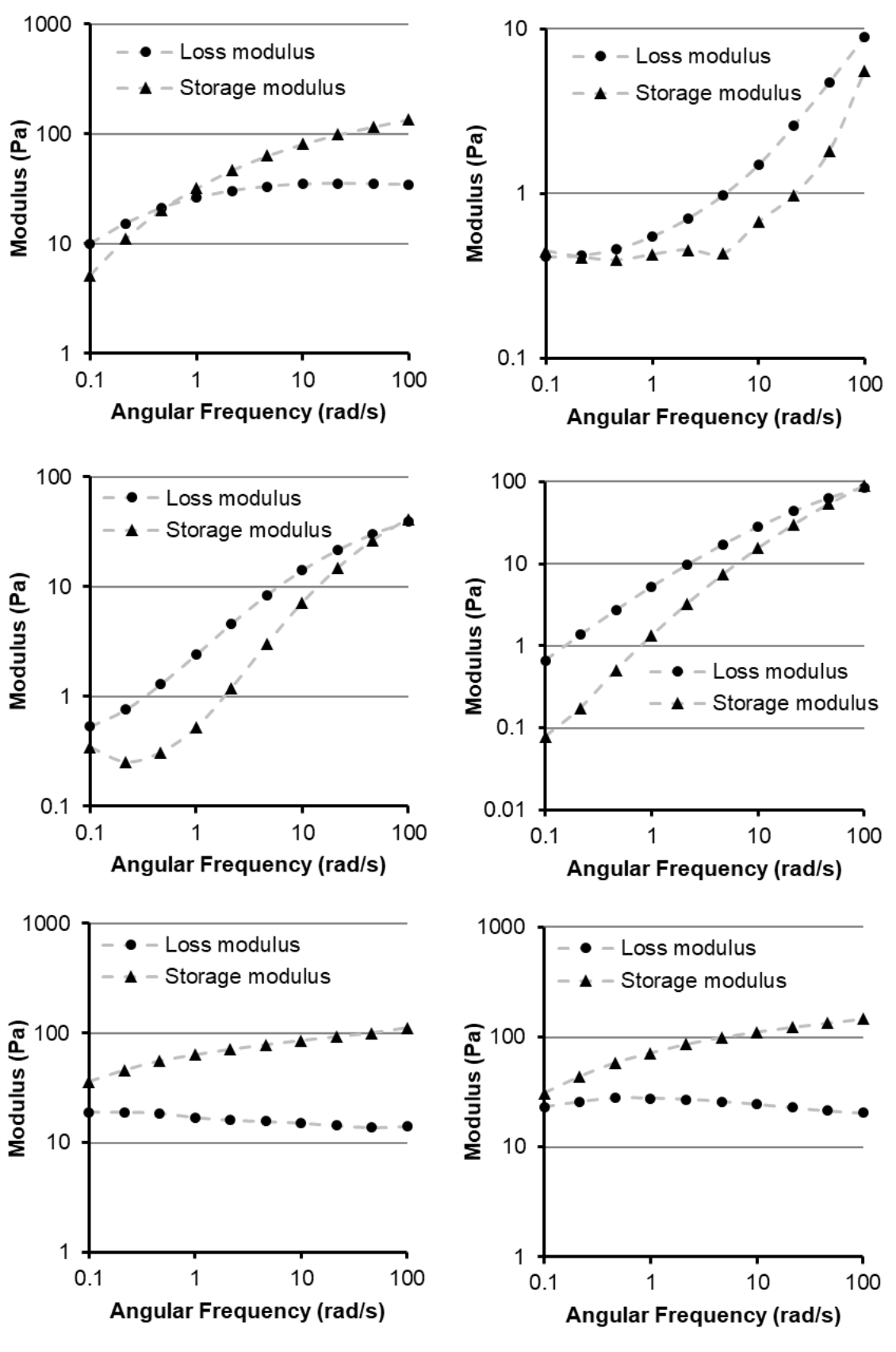

Supplement: S2 Fig — A cross-over frequency was not evident within the operating conditions for all of the formulations. (TIF) [file pone.0216702.s002.tif]
